# Supplementary material for: Chiral Recognition of Hexahelicene on a Surface via the Forming of Asymmetric Heterochiral Trimers
Source: Int J Mol Sci. 2019 Apr 24;20(8):2018. doi: 10.3390/ijms20082018 (PMC6515564; doi:10.3390/ijms20082018)
Supplement: Supplementary file 1 [file ijms-20-02018-s001.pdf]

International Journal of
Molecular Sciences

Communication

Chiral Recognition of Hexahelicene on a Surface via the Forming of Asymmetric Heterochiral Trimers

Hong Zhang[1,2,3], Hong Liu[1], Chengshuo Shen[4], Fuwei Gan[1], Xuelei Su[1], Huibin Qiu[4,*] , Bo Yang[1,*] and Ping Yu[1,*]

[1] School of Physical Science and Technology, ShanghaiTech University, 393 Middle Huaxia Road, Pudong, Shanghai 201210, China; zhanghong1@shanghaitech.edu.cn (H.Z.); liuhong@shanghaitech.edu.cn (H.L.); ganfw@shanghaitech.edu.cn (F.G.); suxl@shanghaitech.edu.cn (X.S.)
[2] Shanghai Institute of Ceramics, Chinese Academy of Sciences, Shanghai 200050, China
[3] University of Chinese Academy of Sciences, Beijing 100049, China
[4] School of Chemistry and Chemical Engineering, State Key Lab of Metal Matrix Composites, Shanghai Jiao Tong University, Shanghai 200240, China; shenchengshuo@sjtu.edu.cn(C. S)
* Correspondence: yangbo1@shanghaitech.edu.cn (B Y); hbqiu@sjtu.edu.cn (H Q); yuping@shanghaitech.edu.cn (P Y)

Received: 5 April 2019; Accepted: 21 April 2019; Published: 24 April 2019

S1. Proof of no influence of NaCl on the [6]H self-assembly

Since it is much easier to pick up CO on the tip from NaCl island than directly from Au(111) surface, NaCl is required for preparing CO functionalized tip for AFM measurements[1,2]. To avoid NaCl influence on the [6]H self-assembly process, we first evaporate NaCl on the surface, then evaporate the molecules afterwards. Moreover, to prove that NaCl has no influence on the self-assembly of [6]H, the samples without NaCl (Fig. S1a,b) and with NaCl (Fig. S1c,d) are compared in the experiments, which give the same experimental results confirming that evaporating NaCl first on the surface has no influence on the self-assembly process of [6]H.

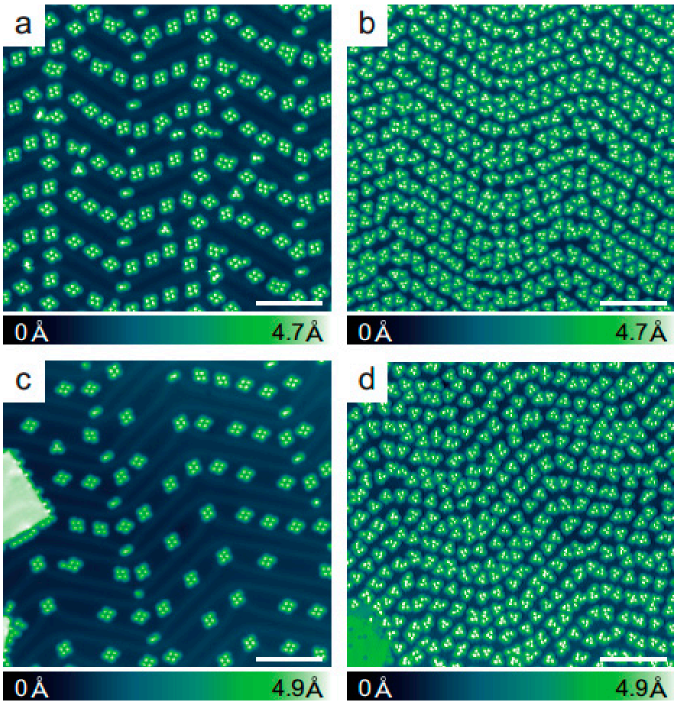

Figure S1. (a,b) Overview STM images of *P*-[6]H and *rac*-[6]H self-assembly on Au(111) without NaCl evaporation. (c,d) Overview STM images of *P*-[6]H and *rac*-[6]H self-assembly on Au(111) with NaCl evaporation before the molecules evaporation. Scale bars: 10nm.

S2. Self-assembly of *P*-[6]H at various coverages

STM images of *P*-[6]H self-assembly at different coverages are displayed in Fig. S2a-d. Tetramers prefer to grow in the fcc regions on the Au(111) surface[3], which is due to the higher adsorption energy of aggregates on fcc regions than on hcp regions[4]. It is shown that for high coverage, the tetramers have been destroyed, there is no well defined self-assembly structure of *P*-[6]H, which could be due to the stress induced by the mismatch between the tetramer and the substrate not allowing the long-range self-assembled structures[5].

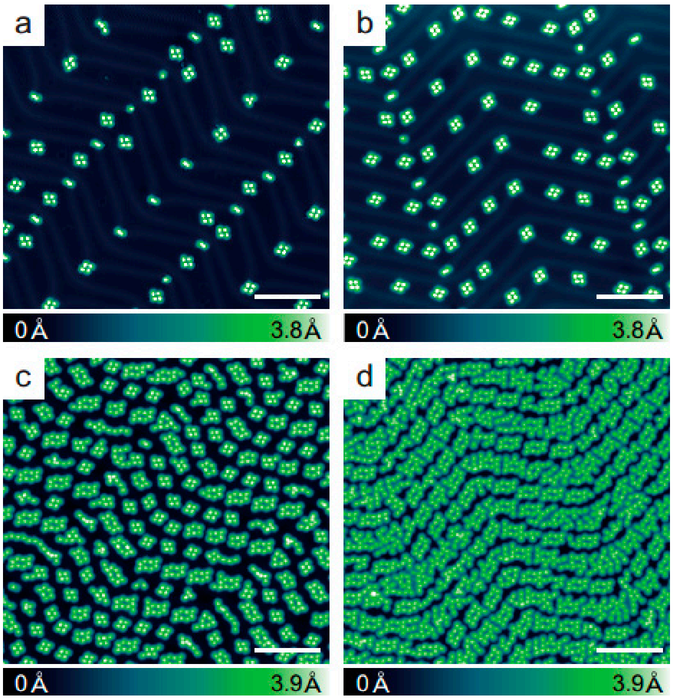

Figure S2. Overview STM images of *P*-[6]H on Au(111) at different coverages. a-d correspond to initial, low, medium and high coverage respectively. Scale bars: 10nm.

S3. Self-assembly of *rac*-[6]H at different coverages

In contrast to *P*-[6]H, asymmetric heterochiral trimers sever as basic building blocks of *rac*-[6]H self-assembly at different coverages.

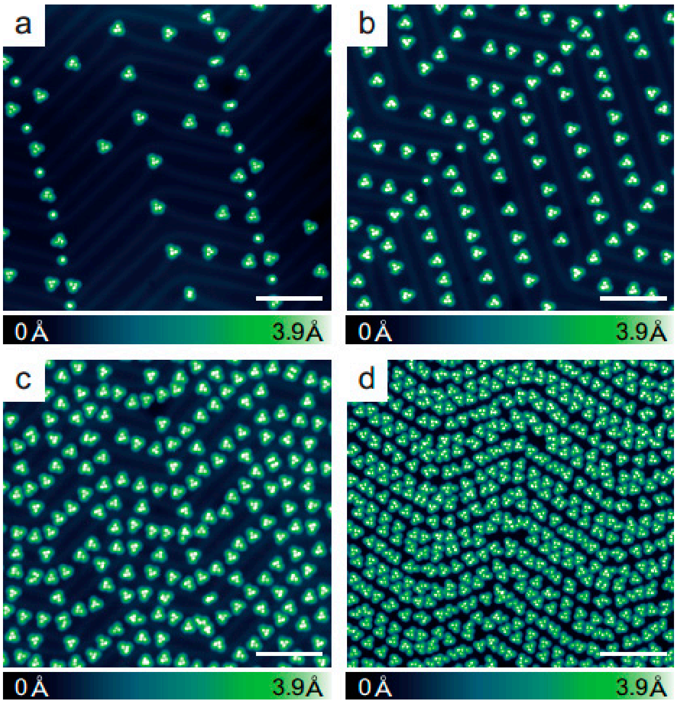

Figure S3. Overview STM images of *rac*-[6]H/Au(111) at different coverages. a-d correspond to initial, low, medium and high coverage respectively. Scale bars: 10nm.

S4. Statistics of separated trimer

To confirm chirality composition of asymmetric trimers further, the same method as described in main text is employed to separate 13 trimers manually, which contains 8 trimer A and 5 trimer B. The upper columns indicate the position before separation and lower columns refer to the same position after separation. Combined STM and nc-AFM measurements points out trimer A is composed of two [6]H with handedness *P* and one enantiomer with handedness *M*, and trimer B owns the opposite chirality composition as M_2P. Hence the conclusion asymmetric trimers are composed of helicenes of both handedness can be drawn.

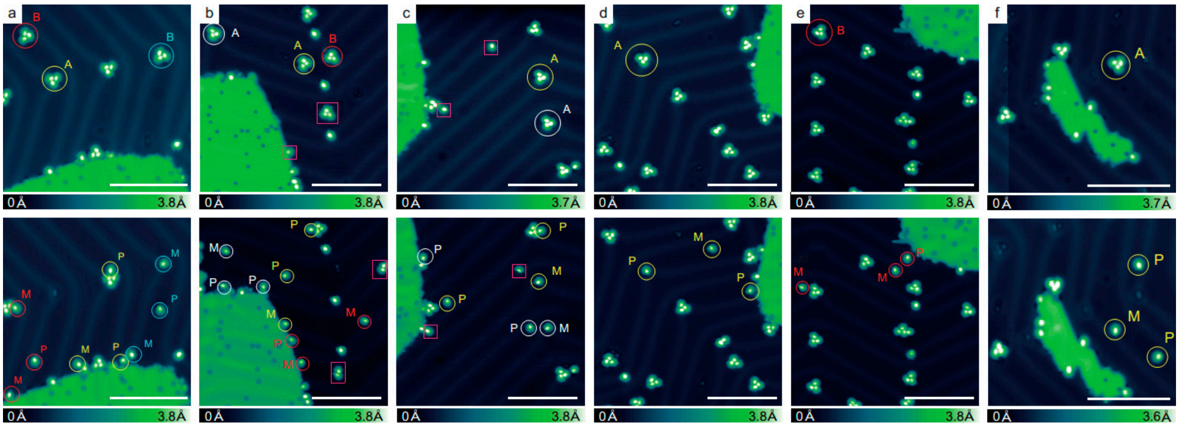

Fig S4. Overview of positions before (upper column) and after (lower column) trimer separation are given. Trimers before separation are marked in colorful circles and helicenes separated from trimer are marked in circles of the same color correspondingly. Clusters in rectangles represent helicenes have changed positions from upper column to the lower column. Scale bars: 10nm.

S5. Handedness of the trimers in the chains

Single chains and double chains are formed by trimers and trimer pairs respectively on Au(111) at high coverage. For the trimer pairs in the double chain, about 80% trimer pairs have the opposite handedness, which are mirror images of each other. Detailed statistics are shown in Table S1. Nevertheless, the handedness of the trimers in the chain developed randomly, which are analyzed in detail in the following STM overview images.

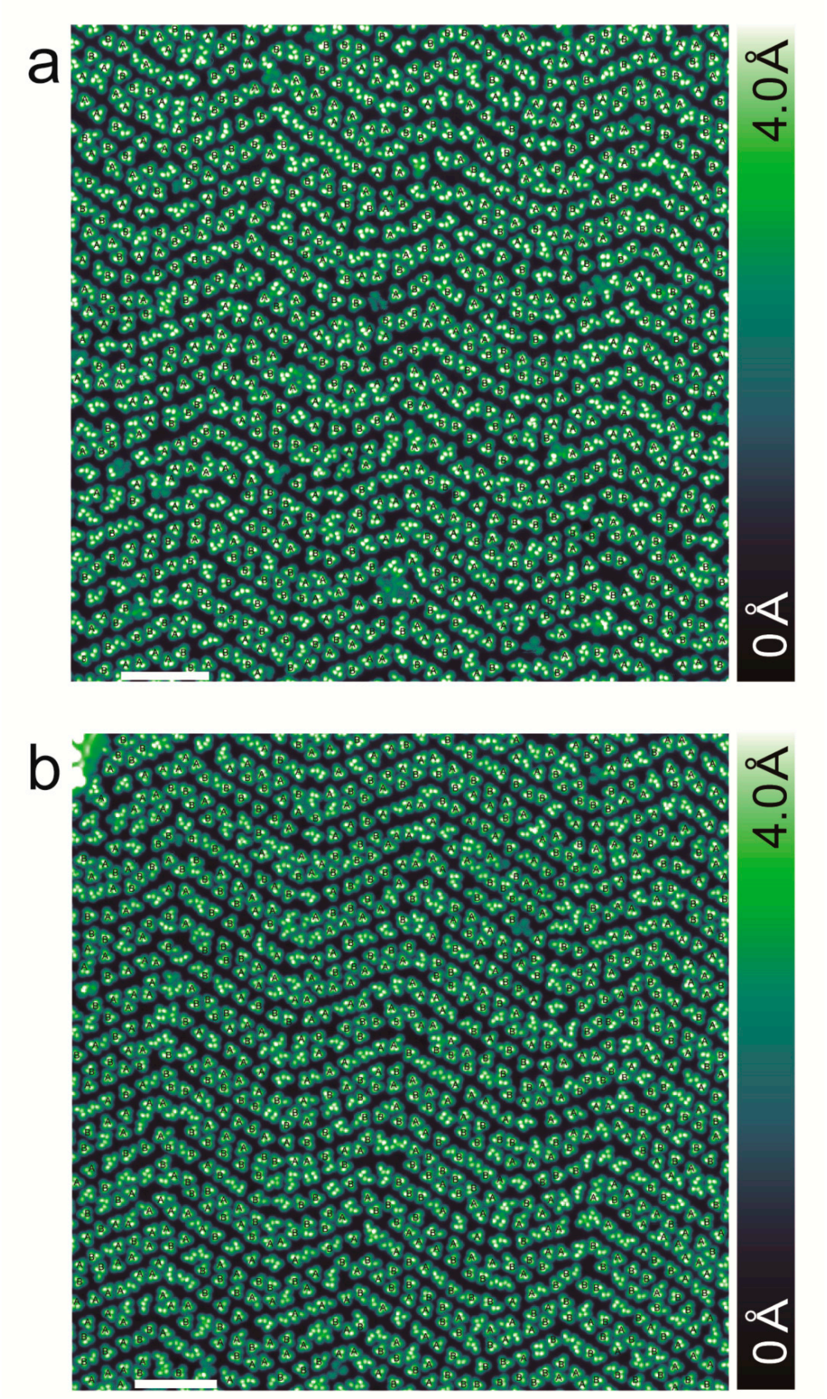

Figure S5. (a,b) STM images of *rac*-[6]H self-assembly at high coverage. Single and double chains alternate on the surface and grow along the herringbone reconstructions. Scale bars: 10nm. (definition of trimer A and B can be found in main text)

Table S1. Handedness of trimer pairs in the double chain[a].

	Homo-trimer pair	Hetero-trimer pair	Total trimer pairs	Hetero-trimer pair rate(%)
S5a	19	83	102	81.4
S5b	31	122	153	79.7
total	50	205	255	80.4

a:Statistics are made from Fig. S5

References

1. Dienel, T.; Kawai, S.; Sode, H.; Feng, X.; Mullen, K.; Ruffieux, P.; Fasel, R.; Groning, O. Resolving Atomic Connectivity in Graphene Nanostructure Junctions. *Nano Lett.* **2015**, *15*, 5185-5190.
2. Su, X.; Xue, Z.; Li, G.; Yu, P. Edge State Engineering of Graphene Nanoribbons. *Nano Lett.* **2018**, *18*, 5744-5751.
3. Chaunchaiyakul, S.; Krukowski, P.; Tsuzuki, T.; Minagawa, Y.; Akai-Kasaya, M.; Saito, A.; Osuga, H.; Kuwahara, Y. Self-Assembly Formation of M-Type Enantiomer of 2,13-Bis(hydroxymethyl)[7]-thiaheterohelicene Molecules on Au(111) Surface Investigated by STM/CITS. *J. Phys. Chem. C* **2015**, *119*, 21434-21442.
4. Chen, W.; Madhavan, V.; Jamneala, T.; Crommie, M.F. Scanning tunneling microscopy observation of an electronic superlattice at the surface of clean gold. *Phys. Rev. Lett.* **1998**, *80*, 1469-1472.
5. Seibel, J.; Parschau, M.; Ernst, K.H. From Homochiral Clusters to Racemate Crystals: Viable Nuclei in 2D Chiral Crystallization. *J. Am. Chem. Soc.* **2015**, *137*, 7970-7973.

© 2019 by the authors. Submitted for possible open access publication under the terms and conditions of the Creative Commons Attribution (CC BY) license (http://creativecommons.org/licenses/by/4.0/).
